# Supplementary material for: Do beer and wine respond to price and tax changes in Vietnam? Evidence from the Vietnam Household Living Standards Survey
Source: BMJ Open. 2019 May 5;9(5):e027076. doi: 10.1136/bmjopen-2018-027076 (PMC6502024; doi:10.1136/bmjopen-2018-027076)
Supplement: Author's manuscript [file bmjopen-2018-027076.draft_revisions.pdf]

BMJ Open is committed to open peer review. As part of this commitment we make the peer review history of every article we publish publicly available.

When an article is published we post the peer reviewers' comments and the authors' responses online. We also post the versions of the paper that were used during peer review. These are the versions that the peer review comments apply to.

The versions of the paper that follow are the versions that were submitted during the peer review process. They are not the versions of record or the final published versions. They should not be cited or distributed as the published version of this manuscript.

BMJ Open is an open access journal and the full, final, typeset and author-corrected version of record of the manuscript is available on our site with no access controls, subscription charges or pay-per-view fees (http://bmjopen.bmj.com).

If you have any questions on BMJ Open's open peer review process please email info.bmjopen@bmj.com

BMJ Open

Do Wine and Beer Respond to Price and Tax Changes in Vietnam? Evidence from the Vietnam Household Living Standards Survey

Journal:	*BMJ Open*
Manuscript ID	bmjopen-2018-027076
Article Type:	Research
Date Submitted by the Author:	04-Oct-2018
Complete List of Authors:	Hien, Nguyen Thi Thu; Thuongmai University<br>Toan, Pham Ngoc; Ministry of Labour, Invalids and Social Affairs<br>Chelwa, Grieve; University of Cape Town, Graduate School of Business<br>Ross, Hana; SALDRU Research Affiliate, University of Cape Town,<br>Thu, Le Thi; Healthbridge Foundation of Canada<br>Anh , Pham Thi Hoang; Healthbridge Foundation of Canada
Keywords:	Alcohol Demand, Deaton, Expenditure Survey, Price Elasticity, Vietnam

SCHOLARONE™
Manuscripts

For peer review only

Do Wine and Beer Respond to Price and Tax Changes in Vietnam? Evidence from the Vietnam Household Living Standards Survey

Nguyen Thi Thu Hien[1], Pham Ngoc Toan[2], Grieve Chelwa[3], Hana Ross[4], Le Thi Thu[5], Pham Thi Hoang Anh[5]

[1] Faculty of Economic – Law, Thuongmai University, Hanoi, Vietnam.

2 Center for Information, Strategic Analysis and Forecast, Institute of labor Science & Social Affairs, Ministry of Labor, Invalids and Social Affairs, Hanoi, Vietnam.

[3]Graduate School of Business, University of Cape Town, South Africa.

[4] University of Cape Town, SALDRU and the School of Economics, South Africa.

[5] HealthBridge Foundation of Canada, Vietnam Office, Hanoi, Vietnam.

Corresponding author: Dr. Grieve Chelwa

Email: grievechelwa@gmail.com

For peer review only

ABSTRACT

Objective. To provide the first ever published estimates of the price and income elasticities of demand for beer and wine in Vietnam and thereby contribute to policy initiatives aimed at reducing the dangerous consumption of alcohol.

Methods. We use a linear approximation of Almost Ideal Demand System (AIDS) and data from the Vietnam Household Living Standards Survey for 2010, 2012 and 2014.

Results: We find that the demand for beer and wine in Vietnam is price and income inelastic with the price elasticities ranging between -0.317 and -0.283 and the income elasticities between 0.156 and 0.401. That is, we find that beer and wine consumption decline whenever their respective prices increase and their consumption increases whenever incomes rise.

Conclusions: The results of the study give credence to calls for increased taxation of alcohol products on public health grounds particularly in an environment where incomes have been growing like they have in Vietnam.

Key words: Alcohol Demand; Deaton; Expenditure Surveys; Price elasticity; Vietnam

For peer review only

Strengths and Limitations of this study

- This paper provides the first ever published estimates of the price and income elasticities of demand for beer and wine in Vietnam
- Often, these estimates are provided for broad alcohol categories but we provide separate estimates for beer and wine
- The paper uses the method pioneered by Angus Deaton that exploits spatial variation of beer and wine prices in Vietnam
- A limitation is that we do not provide elasticity estimates for spirits, another important alcohol type consumed in Vietnam
- A second limitation is that we do not provide cross-price elasticities for beer and wine in our analysis

For peer review only

For peer review only - http://bmjopen.bmj.com/site/about/guidelines.xhtml

INTRODUCTION

The use of alcohol in Vietnam measured in pure alcohol equivalent per person increased significantly from 3.8 litters in 2003-2005 to 6.6 litters in 2008-2010 (1). In 2013, Vietnam consumed 3.0 billion liters of beer and was ranked as number one in Southeast Asia in beer consumption (2).

Excessive beer and wine consumption is not only harmful to health, it also increases domestic violence and traffic accidents. Approximately 33.7% of family violence cases in Vietnam are caused by the use of alcohol (3), while 36.2% and 0.7% of traffic accidents involving men and women were related to alcohol, respectively (4). About 36% of motorcyclists and 66.8% of car drivers involved in the 18,412 traffic accidents from July 2009 to October 2010 had higher than allowed level of blood alcohol concentration (5).

To reduce the harmful effects of the use of alcohol, it is important to implement measures to reduce their consumption (6). Evidence from many countries demonstrates that tax increases resulting in higher prices are one of the most effective measures to control the consumption of alcohol and the associated harms (7, 8). Gallet (2007) assessed the size of the price and income effects on alcohol demand by conducting a meta-analysis of 132 international studies and concluded that the price elasticities of beer and wine were about -0.83 and -1.11, respectively while the income elasticities of beer and wine were about 0.2 and 0.71, respectively (9). He concluded that the estimated responsiveness to price depends on the choice of demand functions, the model, the estimation method and the type of data used. He pointed out that nonlinear demand functions tend to generate smaller elasticities compared to linear demand functions. Bundit et al. (2013) summarized results of 12 studies estimating the demand for alcohol, including studies conducted in China, India, the Philippines, Taiwan and Thailand. The price elasticities of total alcohol ranged from - 0.02 in the Philippines to - 1.53 in China (10).

To assess the impact of tax policy on alcohol consumption in Vietnam, it is important to estimate price and income elasticities of alcohol demand, which indicates the level of consumers' sensitivity to price and income changes. This study is the first to estimate these elasticities for beer and wine in Vietnam using the Vietnam Household Living Standards Surveys of 2010, 2012 and 2014.

For peer review only - http://bmjopen.bmj.com/site/about/guidelines.xhtml

METHOD

The description of the method given here borrows heavily from Chelwa (2015) (11). We used data from the nationally representative Vietnam Household Living Standards Survey (VHLSS) for 2010, 2012 and 2014. Each wave includes data on household income, expenditure and other household characteristics for 9,399 households in 3,133 clusters. In this study we focused on the quantity and the monetary value of beer and wine bought or exchanged in the previous month.

Table 1 reports summary statistics for the variables used in the study. For the period 2010-2014, about 50% and 20% of households consumed wine and beer, their share of the budget allocated to these goods reached 4.5 % and 6.5 %, and the reported average unit value, a proxy for price, was 17,4 thousand VND and 19,7 thousand VND, respectively.

For peer review only

Table 1: VHLSS Summary Statistics

	Variable	Mean	Std. Dev.	Mean	Std. Dev.	Mean	Std. Dev.	Mean	Std. Dev.
		2010		2012		2014		2010-2014	
Average age of head hh	Age	48.3	14.2	49.7	14.2	50.7	14.1	49.6	14.2
Sex of head hh (1-Male, 0-Female)	Sex	0.8	0.4	0.8	0.4	0.7	0.4	0.7	0.4
Average HH size	Size	3.9	1.6	3.9	1.6	3.8	1.6	3.9	1.6
HH with positive wine expenditure	D_v7	0.5	0.5	0.5	0.5	0.4	0.5	0.5	0.5
HH with positive beer expenditure	D_v8	0.2	0.4	0.2	0.4	0.2	0.4	0.2	0.4
Average wine expenditure	v7	38.4	56.4	46.7	61.5	51.1	48.8	45.2	56.3
Average beer expenditure	v8	98.7	111.9	117.4	108.6	140.5	133.9	118.1	119.6
Average unit value wine expenditure	unit_vq7	14.2	31.1	18.2	34.7	20.1	15.2	17.4	28.8
Average unit value beer expenditure	unit_vq8	16.1	10.2	19.9	9.7	23.6	12.0	19.7	11.1
Average HH expenditure	expend	1,054.9	769.7	1,447.7	1,002.3	1,508.6	1,037.6	1,337.0	965.2
Share of total hh expenditure dedicated to wine	w_v7	0.049	0.062	0.042	0.048	0.044	0.053	0.045	0.055
Share of total hh expenditure dedicated to beer	w_v8	0.068	0.062	0.060	0.057	0.067	0.056	0.065	0.058

Source: Calculated from VHLSS

For peer review only

Given that we only have cross-section household data without prices and that the same alcohol tax is applied across Vietnam, we applied the Almost Ideal Demand System (AIDS) model (12) and a unit value equation (12, 13) that allows us to estimate the demand for a product j as a function of its own price, the general price level (pj, P) and income (x) with an identifying assumption that the prices vary at the cluster level:

$$w_i = \alpha_i + \sum_j \gamma_{ij} \ln p_j + \beta_i \ln(\frac{x}{P}) \qquad (1)$$

Where,

$$\ln P = \alpha_0 + \sum_i \alpha_i \ln p_i + \frac{1}{2}\sum\sum \gamma_{ij} \ln p_i \ln p_j \qquad (2)$$

subject to the following constraints:

$$\sum_{i=1}^{n} \alpha_i = 1, \quad \sum_{i=1}^{n} \gamma_{ij} = 0, \quad \sum_{i=1}^{n} \beta_i = 0, \quad \sum_j \gamma_{ij} = 0$$

The "unit values", a proxy for price are calculated as:

$$v_{ic} = x_{ic}/q_{ic} \qquad (3)$$

Where v_{ic}, x_{ic} and q_{ic} are respectively the unit value, expenditure and quantity of wine/beer by household *i* living in commune *c*. However, unit values are not the same thing as price: they hide quality heterogeneity and are subject to measurement error.

To address this weakness, we first conducted an Analysis of Variance (Anova) exercise to divide the total variation in unit values into "within cluster variation" and "between cluster variation". A large F-statistic allowed us to conclude that unit values (prices) vary across geographical areas. Then, we estimated "within cluster" regressions:

$$\ln v_{ic} = \lambda + \beta \ln x_{ic} + \gamma Z_{ic} + \psi \ln\pi_c + e_{ic} \qquad (4) \text{ and}$$

$$w_{ic} = \alpha + \varepsilon \ln x_{ic} + \delta Z_{ic} + \theta \ln\pi_c + (fe_c + u_{ic}) \qquad (5)$$

Where w_{ic} is the share of wine/beer expenditure in total household expenditure for household *i* in cluster *c* and lnv_{ic} is the log of the unit value, derived according to equation (3) for household *i* in cluster *c*, $\ln x_{ic}$ is the log of total household monthly

expenditure, Z_{ic} is a vector of household characteristics (household size, gender of household head, age of household head, years of schooling of the household head, employment status of the household head, urban/rural status, ethnicity of the household head), fe_c is a cluster fixed effect, and u_{ic} and e_{ic} are error terms. $\ln\pi_c$ are the unobserved prices and consequently, equations (4) and (5) are estimated without them.

The third step involves stripping the household level demand and unit values of the effects of household expenditure and household characteristics and then averaging across clusters:

$$\hat{y}^1_c = \frac{1}{nc}\sum_{i=1}(\ln v_{hc} - \hat{\beta}\ln x_{ic} - \hat{\gamma}Z_{ic}) \qquad (6)$$

and

$$\hat{y}^2_c = \frac{1}{nc}\sum_{i=1}(w_{ic} - \hat{\varepsilon}\ln x_{hc} - \hat{\delta}Z_{ic}) \qquad (7)$$

Where *nc* is number of households in cluster c, $\hat{y}^1_c$ and $\hat{y}^2_c$ are the estimates of, respectively, cluster average unit value and cluster average demand after removing the effects of household expenditure and household characteristic.

The next step involves regressing cluster level demand, $\hat{y}^2_c$, on cluster level unit values, $\hat{y}^1_c$. The coefficient on $\hat{y}^1_c$ in such a regression can be obtained by dividing the covariance between $\hat{y}^2_c$ and $\hat{y}^1_c$ by the variance of $\hat{y}^1_c$:

$$\hat{\phi} = \frac{\mathrm{Cov}\left(\widehat{y^2_c},\widehat{y^1_c}\right) - \frac{\widehat{\sigma^{12}}}{n_{size}}}{\mathrm{Var}\left(\widehat{y^1_c}\right) - \frac{\widehat{\sigma^{11}}}{n_{size}}} \qquad (8)$$

where n_{size} is the number of households in a cluster, $\widehat{\sigma^{12}}$ is the estimate of the covariance of the error terms in equations (4) and (5); $\widehat{\sigma^{11}}$ is the variance of the error term in equation (4).

The final step applies quality correction formulas in obtaining the estimate of the price elasticity of demand, $\widehat{\varepsilon_p}$:

$$\widehat{\varepsilon p} = (\hat{\theta}/w) - \hat{\psi} \qquad (9)$$

For peer review only

Where w is the average share of total household expenditure dedicated to wine/beer in the sample, and $\hat{\psi}$ and $\hat{\theta}$ are the estimates of the coefficients on the unobserved price terms in equations (4) and (5), respectively. $\hat{\psi}$ and $\hat{\theta}$ are recovered as follows:

$$\hat{\psi} = 1 - \frac{\hat{\beta}(w-\hat{\theta})}{\hat{\varepsilon}+w} \qquad (10) \text{ and}$$

$$\hat{\theta} = \frac{\hat{\phi}}{1+(w-\hat{\phi})\hat{\xi}} \qquad (11) \text{ with}$$

$$\hat{\xi} = \frac{\hat{\beta}}{\hat{\varepsilon}+w(1-\hat{\beta})} \qquad (12)$$

where $\hat{\beta}$ is the estimate of the coefficient on total household expenditure in equation (4), the within cluster unit value equation, and $\hat{\varepsilon}$ is the coefficient on total household expenditure in equation (5), the within cluster demand equation.

The expenditure elasticities of demand $\hat{\varepsilon_e}$ is calculated according to Deaton (1990) (14):

$$\hat{\varepsilon_e} = 1 + \left(\frac{\hat{\varepsilon}}{w}\right) - \hat{\beta} \qquad (13)$$

We estimate elasticities separately for each VHLSS wave as well for the pooled sample.

The standard errors for the elasticity estimates were obtained by bootstrapping when we run the cluster-level regressions 1000 times resampling the data from an approximating distribution with replacement.

Patients and public involvement

Patients and the public were not involved in this study.

RESULTS

The results of the ANOVA exercise are discussed here but not reported for reasons of space. They are, however, available from the authors upon request. The R squared from the ANOVA exercise indicates that at least 85% and 87% of the variation in unit values for wine and for beer respectively within a wave is attributable to between cluster variation. The F statistics and the associated p values allow us to reject the null hypothesis of no spatial variation in 2010, 2012, 2014 and pooled data.

For peer review only - http://bmjopen.bmj.com/site/about/guidelines.xhtml

The results of the unit value regression, also not reported here for reasons of space, show that households with higher expenditure report higher unit values. In other words, quality plays a role in determining the price. The expenditure elasticities of quality based on individual waves range between 0.06 and 0.07 for wine and between 0.22 and 0.32 for beer. This means that the reported unit values of wine and beer rise by 0.6% - 0.7% and by 2.2% - 3.2% for every 10% increase in household expenditure, respectively.

Households with older household heads and household head with more schooling report higher unit value for wine (not reported here). The impact of household size is negative, meaning that larger households report lower unit values for both wine and beer. Households living in urban areas tend to have higher unit values for both alcohol products while ethnic minorities buy cheaper wine.

Results of regressing wine and beer budget shares on household expenditure and other household characteristics with cluster fixed effects (equation 5) are not reported here but discussed below (for reasons of space).

The results show that there is a negative and statistically significant relationship between household expenditure and the share of the household budget allocated to wine and beer: when household expenditure rise, the budget shares dedicated to wine and beer tend to fall. More years of schooling among the household heads is related to a smaller budget share on both wine and beer. Households with a male HH head, urban and ethnic minority households devote larger budget shares to wine.

The price elasticity estimates ($\hat{\varepsilon}_p$) for 2010, 2012, 2014 and the pooled sample along with bootstrapped standard errors are presented in Table 2.

For peer review only - http://bmjopen.bmj.com/site/about/guidelines.xhtml

Table 2: Estimates of Price elasticity of Demand for Wine and Beer

	Observed Coef.	Bootstrap Std. Err.	z	P>z	Normal-based [95% Conf. Interval]	
**Wine**						
2010	-0.237	0.085	-2.800	0.005	-0.403	-0.071
2012	-0.491	0.029	-16.950	0.000	-0.548	-0.434
2014	-0.359	1.409	-0.260	0.799	-3.122	2.403
Pooled	-0.317	0.030	-10.560	0.000	-0.376	-0.258
**Beer**						
2010	-0.251	0.025	-10.140	0.000	-0.300	-0.203
2012	-0.305	0.032	-9.500	0.000	-0.368	-0.242
2014	-0.346	0.029	-11.850	0.000	-0.404	-0.289
Pooled	-0.283	0.014	-20.120	0.000	-0.311	-0.256

Source: Calculated from VHLSS

For peer review only

All estimates are statistically significant at the 1% level except for 2014 for wine. The price elasticity of wine demand ranges from -0.237 to -0.491 with the pooled estimate centering on -0.317. This means that the demand for wine in Vietnam is expected to decline by 3.17% for every 10% rise in wine prices. The price elasticity of beer demand ranges from -0.251 to -0.346 with the pooled estimate centering on -0.283. This means that the demand for beer is expected to decline by 2.83% for every 10% rise in beer prices. These are conditional price elasticities measuring price responsiveness among households whose members continue to consume wine and beer after a price increase.

The estimates of the expenditure elasticities (ε_e) for 2010, 2012, 2014 in Table 3 show that wine and beer are normal goods in Vietnam. That is, wine and beer demand increases with an increase in household expenditure (income).

For peer review only - http://bmjopen.bmj.com/site/about/guidelines.xhtml

Table 3. Estimates of the expenditure elasticity of demand

	Observed Coef.	Bootstrap Std. Err.	z	P>z	Normal-based [95% Conf. Interval]	
**Wine**						
2010	0.122	0.065	1.890	0.059	-0.005	0.249
2012	0.339	0.040	8.550	0.000	0.261	0.416
2014	0.097	0.080	1.210	0.225	-0.060	0.255
Pooled	0.156	0.034	4.630	0.000	0.090	0.222
**Beer**						
2010	0.484	0.060	8.000	0.000	0.365	0.602
2012	0.406	0.101	4.000	0.000	0.207	0.604
2014	0.503	0.053	9.420	0.000	0.398	0.607
Pooled	0.401	0.036	11.110	0.000	0.330	0.472

Source: Calculated from VHLSS

As in Table 2, all estimates are statistically significant at the 1% level except for the 2014 wine equation. For the pooled data, the expenditure elasticities are 0.156 and 0.401 for wine and beer, respectively, meaning that their consumption of those products goes up as household expenditure/income increases. A 10% increase in expenditure (income) will lead to 1.6% and 4.0% increase in the demand for wine and beer, respectively.

DISCUSSION

We found that price elasticities of demand for beer and wine are negative while their income elasticities are positive meaning that these products are normal goods in Vietnam. The elasticities' magnitudes are lower compared to the average price elasticities reported by (9), but in line with estimates from other Asian countries presented in (10).

The household budget size and living in urban area have a positive impact on the quality of wine and beer consumed. The size of household reduces the unit price, meaning that larger households buy lower quality beer and wine.

For peer review only - http://bmjopen.bmj.com/site/about/guidelines.xhtml

The share of households' expenditures dedicated to wine and beer declines as the total expenditures increase and when the HH head is more educated. Households with a male head, households living in urban areas and ethnic minority households tend to devote larger budget share to wine. The interpretation of these results is complicated by the large variety of wine prices in Vietnam where low-priced wine is considered a cheap alcoholic drink. Our results show that ethnic minorities prefer cheap wine, while those living in urban areas and those with more education buy more expensive wine.

We conclude that the demand for wine and beer in Vietnam is responsive to price: 10% increase in price of wine and beer will result in 3.2% and 2.8% decline in the demand for wine and beer, respectively. This is good news for policy makers who want to use alcohol tax policy to curb the alcohol consumption in Vietnam. However, the tax policy needs to take into account the positive impact of expenditure/income on alcohol consumption – a 10% increase in income will increase the demand for wine and beer by 1.6% and 4.0%, respectively. This means that the government should consider changes in the overall affordability of alcohol products when designing its alcohol tax policy.

For peer review only

Contributors:

PNT did the analysis with the help of GC. NTTH, HR, LTT and PTHA helped with the writing of the manuscript.

Funding:

This work was supported by the International Development Research Centre grant number 107200-002.

Competing Interests: None

Provenance and peer review: Not commissioned; externally peer reviewed

Data sharing statement: All datasets are publicly available

References

1. Ministry of Industry and Trade (MOIT). The evaluation report for the construction industry development planning Beer – Wine – Beverage in Vietnam to 2015, vision 2025. 2008.
2. V.Quyen. Vietnamess people consume more than 3 billion liter of beer and approximate 68 million litter wine. Vietnamnet Electronic Media. 2014.
3. GSO. Vietnam National Survey of Family Violence. 2010.
4. WHO. Global status report on alcohol and health 2014. Geneva 2014.
5. WHO, VIHEMA. Survey in Vietnam from 7/2009-10/2010. 2010.
6. Edwards, G. PA, T. F. Babor, S. Casswell, R. Ferrence, N. Giesbrecht, et al. Alcohol Policy and the Public Good. Oxford: Oxford University Press. 1994.
7. Anderson P, Chisholm D, Fuhr D. Effectiveness and cost-effectiveness of policies and programmes to reduce the harm caused by alcohol. Lancet. 2009;373:2234–46.
8. Babor T, Caetano R, Casswell S, Edwards G, Giesbrecht N, Graham K, et al. Alcohol: No ordinary commodity. Research and public policy (2nd ed.). Oxford, England: Oxford University Press.: 2010.
9. Craig A, Gallet. The demand for alcohol: a meta-analysis of elasticities. The Australian Journal of Agricultural and Resource Economics. 2007;51:121-35.
10. Bundit S., Kevin S., Joanna C., Robert S., Jürgen R. Elasticity of alcohol consumption, alcohol-related harms, and drinking initiation in low- and middle-income countries: A systematic review and meta-analysis. International Journal of Alcohol and Drug Research. 2013;2(1):45-58.
11. Chelwa G. The economics of tobacco control in some African countries 2015. PhD Thesis, The University of Cape Town.
12. Deaton A. S., J. Muellbauer. An Almost Ideal Demand System. American Economics Review. 1980a;70:312-26.
13. S.J.Praisand, H.S.Houthakker. The Analysis of Family Budget. Cambridge University Press Cambridge 1955.
14. Deaton. Price Elasticity From Survey Data: Extensions and Indonesian Results. Journal of Econometrics. 1990.

For peer review only - http://bmjopen.bmj.com/site/about/guidelines.xhtml

For peer review only

For peer review only - http://bmjopen.bmj.com/site/about/guidelines.xhtml

BMJ Open

Do Beer and Wine Respond to Price and Tax Changes in Vietnam? Evidence from the Vietnam Household Living Standards Survey

Journal:	*BMJ Open*
Manuscript ID	bmjopen-2018-027076.R1
Article Type:	Research
Date Submitted by the Author:	17-Jan-2019
Complete List of Authors:	Chelwa, Grieve; University of Cape Town, Graduate School of Business<br>Toan, Pham Ngoc; Ministry of Labour, Invalids and Social Affairs<br>Hien, Nguyen Thi Thu; Thuongmai University<br>Thu, Le Thi; Healthbridge Foundation of Canada<br>Anh , Pham Thi Hoang; Healthbridge Foundation of Canada<br>Ross, Hana; SALDRU Research Affiliate, University of Cape Town,
**Primary Subject Heading**:	Health economics
Secondary Subject Heading:	Health policy
Keywords:	Alcohol Demand, Deaton, Expenditure Survey, Price Elasticity, Vietnam

SCHOLARONE™
Manuscripts

Do Beer and Wine Respond to Price and Tax Changes in Vietnam? Evidence from the Vietnam Household Living Standards Survey

Grieve Chelwa[1], Pham Ngoc Toan[2], Nguyen Thi Thu Hien[3], Le Thi Thu [4], Pham Thi Hoang Anh [4], Hana Ross [5]

[1] Graduate School of Business, University of Cape Town, South Africa.

[2] Center for Information, Strategic Analysis and Forecast, Institute of labor Science & Social Affairs, Ministry of Labor, Invalids and Social Affairs, Hanoi, Vietnam.

[3] Faculty of Economic – Law, Thuongmai University, Hanoi, Vietnam.

[4] HealthBridge Foundation of Canada, Vietnam Office, Hanoi, Vietnam.

[5] SALDRU and the School of Economics, University of Cape Town, South Africa.

Corresponding author: Dr. Grieve Chelwa

Email: grievechelwa@gmail.com

For peer review only

ABSTRACT

Objective. To provide the first ever published estimates of the price and expenditure elasticities of demand for beer and wine in Vietnam and thereby contribute to policy initiatives aimed at reducing the excessive consumption of alcohol.

Methods. We use a linear approximation of Almost Ideal Demand System (AIDS) and data from the Vietnam Household Living Standards Survey for 2010, 2012 and 2014.

Results: We find that the demand for beer and wine in Vietnam is price and expenditure inelastic with average price elasticities of -0.283 and -0.317 and average expenditure elasticities of 0.401 and 0.156, respectively. That is, we find that beer and wine consumption decline whenever their respective prices increase and their consumption increases whenever expenditure rises.

Conclusions: The results of the study lend confidence to calls for increased taxation of alcoholic products on public health grounds in Vietnam.

Key words: Alcohol Demand; Deaton; Expenditure Surveys; Price elasticity; Vietnam

For peer review only

Strengths and Limitations of this study

- This paper provides the first ever published estimates of the price and expenditure elasticities of demand for beer and wine in Vietnam
- Often, these estimates are provided for broad alcohol categories but we provide separate estimates for beer and wine
- The paper uses the method pioneered by Angus Deaton that exploits spatial variation of beer and wine prices in Vietnam
- A limitation is that we do not provide elasticity estimates for spirits, another important alcohol type consumed in Vietnam
- A second limitation is that we do not provide cross-price elasticities for beer and wine in our analysis

For peer review only

For peer review only - http://bmjopen.bmj.com/site/about/guidelines.xhtml

INTRODUCTION

The use of alcohol in Vietnam measured in pure alcohol equivalent per person increased significantly from 3.8 litters in 2003-2005 to 6.6 litters in 2008-2010 (1). Excessive beer and wine consumption is not only harmful to health, it also increases domestic violence and traffic accidents. Approximately 33.7% of family violence cases in Vietnam are caused by the use of alcohol (2), while 36.2% and 0.7% of traffic accidents involving men and women were related to alcohol, respectively (3). About 36% of motorcyclists and 66.8% of car drivers involved in the 18,412 traffic accidents from July 2009 to October 2010 had higher than allowed level of blood alcohol concentration (4).

To reduce the harmful effects of the excessive use of alcohol, it is important to implement measures to reduce their consumption (5). Evidence from many countries demonstrates that tax increases resulting in higher prices are one of the most effective measures to control the excessive consumption of alcohol and the associated harms (6, 7). Gallet (2007) assessed the size of the price and income effects on alcohol demand by conducting a meta-analysis of 132 international studies and concluded that the price elasticities of beer and wine were about -0.83 and -1.11, respectively while the income elasticities were about 0.2 and 0.71, respectively (8). He concluded that the estimated responsiveness to price depends on the choice of demand functions, the model, the estimation method and the type of data used. He pointed out that nonlinear demand functions tend to generate smaller elasticities compared to linear demand functions. Bundit et al. (2013) summarized results of 12 studies estimating the demand for alcohol, including studies conducted in China, India, the Philippines, Taiwan and Thailand. The price elasticities of total alcohol ranged from - 0.02 in the Philippines to - 1.53 in China (9).

To assess the impact of tax policy on alcohol consumption in Vietnam, it is important to estimate price and income elasticities of alcohol demand, which indicate the level of consumers' sensitivity to price and income changes. In this paper we proxy for income using expenditure and therefore estimate expenditure elasticities of demand. Our study focuses on beer and wine, given that together they constitute much of the alcohol consumed in Vietnam. This study is the first to estimate elasticities for beer and wine in Vietnam using the Vietnam Household Living Standards Surveys (VHLSS) of 2010, 2012 and 2014.

For peer review only - http://bmjopen.bmj.com/site/about/guidelines.xhtml

METHOD

The description of the method given here borrows from Chelwa (2015) (10). We use data from the nationally representative Vietnam Household Living Standards Survey (VHLSS) for 2010, 2012 and 2014. The VHLSS is conducted by the General Statistics Office (GSO) in Vietnam and informs much of the country's planning processes. Each wave includes data on household income, expenditure and other household characteristics for 9,399 households in 3,133 clusters (or communes as they are known in Vietnam). A commune is the lowest administrative unit in Vietnam. We focus on the quantity and the monetary value of beer and wine bought in the previous month.

Given that we only have cross-sectional household data without prices and that the same alcohol tax is applied across Vietnam, we apply the Almost Ideal Demand System (AIDS) (11) and a unit value equation (11, 12) to estimate elasticities. The main identifying assumption is that prices vary at the cluster level, which in Vietnam is at the commune level. This assumption is reasonable given that households that are geographically proximate, i.e. in the same commune, face similar prices in Low and Middle Income countries (LMICs). This assumption generates exogenous variation in prices which is then used to estimate the elasticities.

Given that the VHLSS does not have price information, we proxy for prices using "unit values" calculated as:

$$v_{ic} = \frac{x_{ic}}{q_{ic}} \qquad (1)$$

Where v_{ic} ,x_{ic} and q_{ic} are respectively the unit value, expenditure and quantity of wine/beer by household *i* living in commune *c*. However, unit values are not the same thing as prices. They hide quality differences and are subject to measurement error. The method used in this paper (described in full below) addresses these shortcomings in using unit values in place of prices.

The method proceeds in a series of steps. First one conducts an Analysis of Variance (ANOVA) exercise to divide the total variation in unit values into "within commune

For peer review only - http://bmjopen.bmj.com/site/about/guidelines.xhtml

variation" and "between commune variation". The ANOVA is conducted to verify our main identifying assumption that prices, unit values in this case, vary at the commune level. A large F-statistic in this exercise leads to the conclusion that unit values vary across communes.

In a second step, one estimates "within commune" regressions of the form:

$$lnv_{ic} = \lambda + \beta lnX_{ic} + \boldsymbol{\gamma Z_{ic}} + \psi ln\pi_c + e_{ic} \quad (2) \; and$$

$$w_{ic} = \alpha + \varepsilon lnX_{ic} + \boldsymbol{\delta Z_{ic}} + \theta ln\pi_c + (FE_c + u_{ic}) \qquad (3)$$

Where w_{ic} is the share of wine/beer expenditure in total household expenditure for household i in commune c and lnv_{ic} is the log of the unit value, derived according to equation (1) for household i in commune c. lnX_{ic} is the log of total household monthly expenditure, $\boldsymbol{Z_{ic}}$ is a vector of household characteristics (household size, gender of household head, age of household head, years of schooling of the household head, employment status of the household head, urban/rural location of the household and ethnicity of the household head), FE_c is a commune fixed effect, and u_{ic} and e_{ic} are the standard regression error terms. Given that the VHLSS does not contain price information, $ln\pi_c$ are the unobserved prices and consequently, equations (2) and (3) are estimated without them. However, the coefficients on the unobserved price terms can be recovered (see below).

A third step involves stripping the household level demand and unit values of the effects of household expenditure and household characteristics and then averaging across communes. The stripping and averaging is done according to the following equations:

$$\widehat{y_c^1} = \frac{1}{n_c}\sum_{i=1}^{n_c}(lnv_{ic} - \hat{\beta} lnx_{ic} - \boldsymbol{\hat{\gamma} Z_{ic}}) \qquad (4)$$

For peer review only - http://bmjopen.bmj.com/site/about/guidelines.xhtml

and $$\widehat{y_c^2} = \frac{1}{n_c}\sum_{i=1}^{n_c}(w_{ic} - \hat{\varepsilon} ln x_{ic} - \hat{\boldsymbol{\delta}}\boldsymbol{Z}_{ic}) \qquad (5)$$

Where n_c is number of households in commune c, $\widehat{y_c^1}$ and $\widehat{y_c^2}$ are the estimates of, respectively, commune average unit value and commune average demand after removing the effects of household expenditure and household characteristics.

The next step involves regressing commune level demand, $\widehat{y_c^2}$, on commune level unit values, $\widehat{y_c^1}$. The coefficient on $\widehat{y_c^1}$ in such a regression can be obtained by dividing the covariance between $\widehat{y_c^2}$ and $\widehat{y_c^1}$ by the variance of $\widehat{y_c^1}$:

$$\hat{\phi} = \frac{Cov\left(\widehat{y_c^2}, \widehat{y_c^1}\right) - \frac{\widehat{\sigma^{12}}}{n_c}}{Var\left(\widehat{y_c^1}\right) - \frac{\widehat{\sigma^{11}}}{n_c^+}} \qquad (6)$$

where n_c^+ is the number of households in a commune reporting positive expenditures on tobacco and n_c is the number of households in a commune; $\widehat{\sigma^{12}}$ is the estimate of the covariance of the errors in equations (2) and (3); $\widehat{\sigma^{11}}$ is the variance of the errors in equation (2). The variance and covariance of the error terms correct for measurement error in unit values.

The final step applies quality correction formulas in obtaining the estimate of the price elasticity of demand, $\widehat{\varepsilon_P}$:

$$\widehat{\varepsilon_P} = \left(\frac{\hat{\theta}}{\overline{\overline{w}}}\right) - \hat{\psi} \qquad (7)$$

Where $\overline{w}$ is the average share of total household expenditure dedicated to wine/beer in the sample, and $\hat{\psi}$ and $\hat{\theta}$ are the estimates of the coefficients on the unobserved price terms in equations (2) and (3), respectively. $\hat{\psi}$ and $\hat{\theta}$ are recovered as follows:

$$\hat{\psi} = 1 - \frac{\hat{\beta}(\overline{w} - \hat{\theta})}{\hat{\varepsilon} + \overline{w}} \qquad (8) \text{ and}$$

$$\hat{\theta} = \frac{\hat{\phi}}{1 + (\overline{w} - \hat{\phi})\hat{\zeta}} \qquad (9) \text{ with}$$

For peer review only - http://bmjopen.bmj.com/site/about/guidelines.xhtml

$$\hat{\zeta} = \frac{\hat{\beta}}{\hat{\varepsilon} + \overline{w}(1 - \hat{\beta})} \qquad (10)$$

where $\hat{\beta}$ is the estimate of the coefficient on total household expenditure in equation (2), the within commune unit value equation, and $\hat{\varepsilon}$ is the coefficient on total household expenditure in equation (3), the within commune demand equation.

The expenditure elasticities of demand $\widehat{\varepsilon_e}$ is calculated according to Deaton (1990) (13):

$$\widehat{\varepsilon_e} = 1 + \left(\frac{\hat{\varepsilon}}{\overline{w}}\right) - \hat{\beta} \qquad (11)$$

We estimate elasticities separately for each VHLSS wave as well for the pooled sample. The standard errors for the elasticity estimates are obtained by the method of the bootstrap.

Table 1 reports some summary statistics for the variables used in the study. The average household budget shares allocated to wine and beer were respectively 4.5 % and 6.5 % over the period under study. The reported average unit values were VND17,400 (approximately USD0.80) for wine and VND19,700 (approximately USD0.92) for beer. VND stands for Vietnamese Dong, Vietnam's currency.

For peer review only - http://bmjopen.bmj.com/site/about/guidelines.xhtml

Table 1: Summary Statistics from the VHLSS

	Mean	*Std. Dev.*	*Mean*	*Std. Dev.*	*Mean*	*Std. Dev.*	*Mean*	*Std. Dev.*
	2010		2012		2014		2010-2014	
Average age of Household Head	48.3	14.2	49.7	14.2	50.7	14.1	49.6	14.2
Gender of Household Head (1 Male, 0 Female)	0.8	0.4	0.8	0.4	0.7	0.4	0.7	0.4
Average Household Size	3.9	1.6	3.9	1.6	3.8	1.6	3.9	1.6
Ethnicity of Household head (1- ethnic minority; 0-otherwise)	0.17	0.38	0.17	0.38	0.17	0.37	0.17	0.37
Average Year of schooling of Household head	7.58	4.40	7.60	4.40	7.74	4.41	7.64	4.41
Location of household (1 – urban, 0 - rural	0.28	0.45	0.29	0.45	0.30	0.46	0.29	0.45
Household head in wage employment (1-yes; 0- no)	0.41	0.49	0.39	0.49	0.40	0.49	0.40	0.49
Average wine expenditure (in thousands of VND)	38.4	56.4	46.7	61.5	51.1	48.8	45.2	56.3
Average beer expenditure (in thousands of VND)	98.7	111.9	117.4	108.6	140.5	133.9	118.1	119.6
Average unit value for wine (in thousands of VND)	14.2	31.1	18.2	34.7	20.1	15.2	17.4	28.8
Average unit value for beer (in thousands of VND)	16.1	10.2	19.9	9.7	23.6	12.0	19.7	11.1
Average household expenditure (in thousands of VND)	1,054.9	769.7	1,447.7	1,002.3	1,508.6	1,037.6	1,337.0	965.2
Share of total household expenditure dedicated to wine	0.049	0.062	0.042	0.048	0.044	0.053	0.045	0.055
Share of total household expenditure dedicated to beer	0.068	0.062	0.060	0.057	0.067	0.056	0.065	0.058

For peer review only - http://bmjopen.bmj.com/site/about/guidelines.xhtml

Source: Calculated from VHLSS. VND stands for Vietnamese Dong.

Patients and public involvement

Patients and the public were not involved in this study.

RESULTS

The results of the ANOVA exercise are discussed here but not reported for reasons of space. They are, however, available from the authors upon request. The R squared from the ANOVA exercise indicates that at least 85% of the variation in unit values for wine and beer is attributable to variations between communes. The F statistics and the associated p values allow us to reject the null hypothesis of no spatial variation in 2010, 2012, 2014 and pooled data. In other words, prices vary significantly between communes in Vietnam.

The results of the unit value regressions (equation 2) are reported in Table 2. The results show that households with higher expenditure report higher unit values. In other words, unit values reflect choices about quality. These so-called expenditure elasticities of quality range between 0.06 and 0.07 for wine and between 0.22 and 0.32 for beer. This means that the reported unit values of wine and beer rise by 0.6% to 0.7% and by 2.2% to 3.2% for every 10% increase in household expenditure, respectively.

Households with older household heads and with heads with more schooling report higher unit values for wine. The impact of household size is negative for both wine and beer, implying that larger households report lower unit values. Households living in urban areas tend to have higher unit values for both alcohol products while ethnic minorities report lower unit values for wine.

For peer review only - http://bmjopen.bmj.com/site/about/guidelines.xhtml

Table 2: Wine and Beer Unit Values Coefficients

	Wine				Beer			
	2010	2012	2014	Pooled	2010	2012	2014	Pooled
VARIABLES	lnunit_v7				lnunit_v8			
LnX	0.060***	0.070***	0.066***	0.137***	0.316***	0.238***	0.213***	0.343***
	(0.009)	(0.010)	(0.011)	(0.006)	(0.021)	(0.022)	(0.021)	(0.012)
Gender	-0.025	-0.018	0.005	-0.003	-0.015	-0.001	0.011	0.008
	(0.016)	(0.016)	(0.018)	(0.010)	(0.027)	(0.027)	(0.026)	(0.016)
LnAge	0.030	0.131***	0.082***	0.118***	-0.066	-0.069	-0.095**	-0.048*
	(0.021)	(0.023)	(0.026)	(0.014)	(0.042)	(0.045)	(0.045)	(0.026)
LnSchool	0.111***	0.155***	0.164***	0.133***	-0.003	-0.008	-0.006	-0.027**
	(0.009)	(0.010)	(0.011)	(0.006)	(0.023)	(0.022)	(0.020)	(0.013)
Employment	0.005	0.025*	-0.002	0.015*	-0.027	-0.014	-0.044*	-0.019
	(0.012)	(0.013)	(0.013)	(0.008)	(0.025)	(0.026)	(0.025)	(0.015)
Ln Size	-0.030**	-0.064***	-0.025	-0.093***	-0.192***	-0.183***	-0.142***	-0.224***
	(0.015)	(0.016)	(0.017)	(0.010)	(0.032)	(0.031)	(0.031)	(0.019)
Urban	0.117***	0.109***	0.137***	0.096***	0.053**	0.115***	0.128***	0.074***
	(0.013)	(0.014)	(0.015)	(0.009)	(0.024)	(0.023)	(0.022)	(0.014)
Ethnic	-0.028*	-0.023	-0.053***	0.011	0.076*	0.050	0.029	0.055**
	(0.015)	(0.015)	(0.017)	(0.010)	(0.040)	(0.044)	(0.048)	(0.026)
Constant	1.851***	1.564***	1.808***	1.170***	0.909***	1.586***	1.971***	0.836***
	(0.102)	(0.114)	(0.129)	(0.069)	(0.215)	(0.236)	(0.242)	(0.134)
Observations	4,241	4,199	3,758	12,198	1,918	1,717	1,705	5,340
R-squared	0.112	0.138	0.151	0.149	0.156	0.120	0.109	0.179

Robust standard errors in parenthesis. LnX (natural logarithm of household expenditure), Gender (gender of household head), LnAge (natural logarithm of age of household head), LnSchool (natural logarithm of years of schooling of household head), Employment (employment status of household head), Urban (location of household), Ethnic (ethnic group of household head). *** p<0.01, ** p<0.05, * p<0.1

Results of regressing wine and beer budget shares on household expenditure and other household characteristics (equation 3) are reported in Table 3. The results show that there is a negative and statistically significant relationship between household expenditure and the share of the household budget allocated to wine and beer. More years of schooling among household heads is related to a smaller budget share on both wine and beer.

For peer review only - http://bmjopen.bmj.com/site/about/guidelines.xhtml

Households with a male head, located in urban areas and from an ethnic minority devote larger budget shares to wine.

Table 3: Beer and Wine Budget Shares

	Wine				Beer			
	2010	2012	2014	Pooled	2010	2012	2014	Pooled
VARIABLES		w_v7				w_v8		
Lnx	-0.037***	-0.027***	-0.038***	-0.032***	-0.013***	-0.023***	-0.018***	-0.017***
	(0.004)	(0.002)	(0.004)	(0.002)	(0.005)	(0.007)	(0.004)	(0.003)
Sex	0.002	0.004**	0.006***	0.004***	0.004	0.003	0.002	0.003*
	(0.003)	(0.002)	(0.002)	(0.001)	(0.004)	(0.003)	(0.003)	(0.002)
Lnage	-0.009***	-0.002	0.001	-0.003	-0.011	-0.006	-0.007	-0.007*
	(0.003)	(0.003)	(0.004)	(0.002)	(0.007)	(0.006)	(0.006)	(0.004)
Lnschool	-0.001	-0.005***	-0.001	-0.002***	-0.005	-0.013***	-0.012***	-0.011***
	(0.001)	(0.001)	(0.001)	(0.001)	(0.003)	(0.003)	(0.003)	(0.002)
EmW	-0.009***	-0.003*	-0.006***	-0.006***	-0.002	-0.000	-0.000	-0.000
	(0.002)	(0.001)	(0.002)	(0.001)	(0.004)	(0.003)	(0.003)	(0.002)
EmSelf	-0.006***	-0.000	-0.002	-0.003***	0.000	0.002	-0.001	0.000
	(0.002)	(0.001)	(0.002)	(0.001)	(0.004)	(0.004)	(0.003)	(0.002)
Lnsize	0.003	-0.003	0.009*	0.002	-0.022***	-0.012**	-0.016***	-0.018***
	(0.003)	(0.003)	(0.004)	(0.002)	(0.006)	(0.005)	(0.005)	(0.003)
Urban	0.004**	0.005***	0.007***	0.004***	0.001	0.008	-0.003	0.002
	(0.002)	(0.002)	(0.002)	(0.001)	(0.004)	(0.005)	(0.003)	(0.003)
Ethnic	0.018***	0.019***	0.018***	0.019***	0.006	0.009	-0.009	0.003
	(0.002)	(0.002)	(0.002)	(0.002)	(0.006)	(0.006)	(0.006)	(0.004)
Constant	0.326***	0.244***	0.292***	0.274***	0.240***	0.296***	0.281***	0.261***
	(0.029)	(0.019)	(0.028)	(0.015)	(0.048)	(0.051)	(0.038)	(0.027)
Observations	4,241	4,199	3,758	12,198	1,918	1,717	1,705	5,340
R-squared	0.253	0.236	0.270	0.247	0.047	0.086	0.086	0.066

Robust standard errors in parenthesis. LnX (natural logarithm of household expenditure), Gender (gender of household head), LnAge (natural logarithm of age of household head), LnSchool (natural logarithm of years of schooling of household head), Employment (employment status of household head), Urban (location of household) and Ethnic (ethnic group of household head). *** p<0.01, ** p<0.05, * p<0.1

The price elasticity estimates, $\widehat{\varepsilon_P}$, for 2010, 2012, 2014 and the pooled sample are presented in Table 4.

For peer review only - http://bmjopen.bmj.com/site/about/guidelines.xhtml

Table 4: Estimates of Price elasticity of Demand for Wine and Beer

	Observed Coef.	Bootstrap Std. Err.	Z	P>z	Normal-based [95% Conf. Interval]	
**Wine**						
2010	-0.237	0.085	-2.800	0.005	-0.403	-0.071
2012	-0.491	0.029	-16.950	0.000	-0.548	-0.434
2014	-0.359	1.409	-0.260	0.799	-3.122	2.403
Pooled	-0.317	0.030	-10.560	0.000	-0.376	-0.258
**Beer**						
2010	-0.251	0.025	-10.140	0.000	-0.300	-0.203
2012	-0.305	0.032	-9.500	0.000	-0.368	-0.242
2014	-0.346	0.029	-11.850	0.000	-0.404	-0.289
Pooled	-0.283	0.014	-20.120	0.000	-0.311	-0.256

Source: Calculated from VHLSS

All estimates are statistically significant at the 1% level except for wine in 2014. The price elasticity of demand for wine ranges from -0.237 to -0.491 with the pooled estimate centering on -0.317. This means that the demand for wine in Vietnam is expected to decline on average by 3.17% for every 10% rise in wine prices. The price elasticity of demand for beer ranges from -0.251 to -0.346 with the pooled estimate centering on -0.283. This means that the demand for beer is expected to decline by 2.83% on average for every 10% rise in beer prices.

The estimates of the expenditure elasticities, $\hat{\varepsilon_e}$, for 2010, 2012, 2014 are reported in Table 5. As in Table 4, all estimates are statistically significant at the 1% level except for wine in 2014. For the pooled data, the expenditure elasticities are 0.156 and 0.401 for wine and beer, respectively. A 10% increase in expenditure leads to a 1.6% and 4.0% increase, on average, in the demand for wine and beer, respectively. This shows that increases in household income, proxied here by household expenditure, will increase wine and beer consumption in Vietnam.

For peer review only - http://bmjopen.bmj.com/site/about/guidelines.xhtml

Table 5. Estimates of the expenditure elasticity of demand

	Observed Coef.	Bootstrap Std. Err.	z	P>z	Normal-based [95% Conf. Interval]	
**Wine**						
2010	0.122	0.065	1.890	0.059	-0.005	0.249
2012	0.339	0.040	8.550	0.000	0.261	0.416
2014	0.097	0.080	1.210	0.225	-0.060	0.255
Pooled	0.156	0.034	4.630	0.000	0.090	0.222
**Beer**						
2010	0.484	0.060	8.000	0.000	0.365	0.602
2012	0.406	0.101	4.000	0.000	0.207	0.604
2014	0.503	0.053	9.420	0.000	0.398	0.607
Pooled	0.401	0.036	11.110	0.000	0.330	0.472

Source: Calculated from VHLSS

DISCUSSION

This paper estimates the first ever price and expenditure elasticities of demand for wine and beer in Vietnam using the VHLSS. The statistical analysis revealed that wine and beer in Vietnam are price inelastic. In other words, the quantity demanded of wine and beer responds less than proportionately to price increases. These results are in line with elasticity estimates from other parts of the world (8).

Our results on the price inelastic nature of the demand for wine and beer has two immediate implications for the authorities in Vietnam. First, it means that tax measures can be used as an effective tool to control the excessive consumption of alcohol in Vietnam. Second, the government can raise additional tax revenue by increasing the level of alcohol taxes in the country.

The results on the expenditure elasticities of demand also have implications for the country. Our estimated elasticities of demand, which are all positive, mean that the quantity demanded of wine and beer in Vietnam increases every time household

For peer review only - http://bmjopen.bmj.com/site/about/guidelines.xhtml

expenditure (or household income) increases. This means that alcohol consumption will continue to increase, and increase at a fast pace, given that Vietnam is a fast growing economy. The gives further impetus to the use of tax measures in curbing excessive demand especially in an environment of fast rising incomes.

Our paper has three limitations, however. First, we are unable to provide elasticity estimates for spirits, another important alcohol category. This is because we do not have good data on spirits to enable us estimate their elasticities. Second, we are unable to provide cross-price elasticities between wine and beer. Cross price elasticities are important because policymakers often want to know how the demand for one good responds to levying a tax on a close substitute. Lastly, our elasticity estimates are conditional elasticity estimates and not total elasticity estimates. In other words, we only provide demand responses for current drinkers of wine and beer and not for the entire population. In other words, we do not estimate total price elasticities. However, empirically, conditional elasticities are often half the absolute value of total elasticities (14). This means that the total demand response to prices is likely to be much higher than the estimates presented in this paper.

Contributors:

PNT did the analysis with the help of GC. NTTH, HR, LTT and PTHA helped with the writing of the manuscript.

Funding:

This work was supported by the International Development Research Centre grant number 107200-002.

Ethics approval: This study did not require ethics approval as it uses the Vietnam Household Living Standards Survey which is a secondary dataset that is publically available

Competing Interests: None

Provenance and peer review: Not commissioned; externally peer reviewed

Data sharing statement: All datasets are publicly available

For peer review only - http://bmjopen.bmj.com/site/about/guidelines.xhtml

References

1. Ministry of Industry and Trade (MOIT). The evaluation report for the construction industry development planning: Beer and Wine Beverage in Vietnam to 2015, vision 2025. 2008.

2. General Statistics Office. Vietnam National Survey of Family Violence. 2010.

3. World Health Organization. Global status report on alcohol and health 2014. Geneva 2014.

4. World Health Organization, Health Environment Management Survey in Vietnam: 2009- 2010. 2010.

5. Edwards, G. PA, T. F. Babor, S. Casswell, R. Ferrence, N. Giesbrecht, et al. Alcohol Policy and the Public Good. Oxford: Oxford University Press. 1994.

6. Anderson P, Chisholm D, Fuhr D. Effectiveness and cost-effectiveness of policies and programmes to reduce the harm caused by alcohol. Lancet. 2009;373:2234–46.

7. Babor T, Caetano R, Casswell S, Edwards G, Giesbrecht N, Graham K, et al. Alcohol: No ordinary commodity. Research and public policy (2nd ed.). Oxford, England: Oxford University Press.: 2010.

8. Craig A, Gallet. The demand for alcohol: a meta-analysis of elasticities. The Australian Journal of Agricultural and Resource Economics. 2007;51:121-35.

9. Bundit S., Kevin S., Joanna C., Robert S., Jürgen R. Elasticity of alcohol consumption, alcohol-related harms, and drinking initiation in low- and middle-income countries: A systematic review and meta-analysis. International Journal of Alcohol and Drug Research. 2013;2(1):45-58.

10. Chelwa G. The economics of tobacco control in some African countries 2015. PhD Thesis, The University of Cape Town.

11. Deaton A. S., J. Muellbauer. An Almost Ideal Demand System. American Economics Review. 1980a;70:312-26.

12. S.J.Praisand, H.S.Houthakker. The Analysis of Family Budget. Cambridge University Press Cambridge 1955.

13. Deaton. Price Elasticity From Survey Data: Extensions and Indonesian Results. Journal of Econometrics. 1990.

For peer review only - http://bmjopen.bmj.com/site/about/guidelines.xhtml

14. International Agency for Research on Cancer. Handbook on the effectiveness of tax and price policies for tobacco control. Geneva: World Health Organization 2011

For peer review only

BMJ Open

Do Beer and Wine Respond to Price and Tax Changes in Vietnam? Evidence from the Vietnam Household Living Standards Survey

Journal:	*BMJ Open*
Manuscript ID	bmjopen-2018-027076.R2
Article Type:	Research
Date Submitted by the Author:	13-Feb-2019
Complete List of Authors:	Chelwa, Grieve; University of Cape Town, Graduate School of Business<br>Toan, Pham Ngoc; Ministry of Labour, Invalids and Social Affairs<br>Hien, Nguyen Thi Thu; Thuongmai University<br>Thu, Le Thi; Healthbridge Foundation of Canada<br>Anh , Pham Thi Hoang; Healthbridge Foundation of Canada<br>Ross, Hana; SALDRU Research Affiliate, University of Cape Town,
**Primary Subject Heading**:	Health economics
Secondary Subject Heading:	Health policy
Keywords:	Alcohol Demand, Deaton, Expenditure Survey, Price Elasticity, Vietnam

SCHOLARONE™
Manuscripts

Do Beer and Wine Respond to Price and Tax Changes in Vietnam? Evidence from the Vietnam Household Living Standards Survey

Grieve Chelwa[1], Pham Ngoc Toan[2], Nguyen Thi Thu Hien[3], Le Thi Thu [4], Pham Thi Hoang Anh [4], Hana Ross [5]

[1] Graduate School of Business, University of Cape Town, South Africa.

[2] Center for Information, Strategic Analysis and Forecast, Institute of labor Science & Social Affairs, Ministry of Labor, Invalids and Social Affairs, Hanoi, Vietnam.

[3] Faculty of Economic – Law, Thuongmai University, Hanoi, Vietnam.

[4] HealthBridge Foundation of Canada, Vietnam Office, Hanoi, Vietnam.

[5] SALDRU and the School of Economics, University of Cape Town, South Africa.

Corresponding author: Dr. Grieve Chelwa

Email: grievechelwa@gmail.com

For peer review only

ABSTRACT

Objective. To provide the first ever published estimates of the price and expenditure elasticities of demand for beer and wine in Vietnam and thereby contribute to policy initiatives aimed at reducing the excessive consumption of alcohol.

Methods. We use a linear approximation of Almost Ideal Demand System (AIDS) and data from the Vietnam Household Living Standards Survey for 2010, 2012 and 2014.

Results: We find that the demand for beer and wine in Vietnam is price and expenditure inelastic with average price elasticities of -0.283 and -0.317 and average expenditure elasticities of 0.401 and 0.156, respectively. That is, we find that beer and wine consumption decline whenever their respective prices increase and their consumption increases whenever expenditure rises.

Conclusions: The results of the study lend confidence to calls for increased taxation of alcoholic products on public health grounds in Vietnam.

Key words: Alcohol Demand; Deaton; Expenditure Surveys; Price elasticity; Vietnam

For peer review only

For peer review only - http://bmjopen.bmj.com/site/about/guidelines.xhtml

Strengths and Limitations of this study

- This paper provides the first ever published estimates of the price and expenditure elasticities of demand for beer and wine in Vietnam
- Often, these estimates are provided for broad alcohol categories but we provide separate estimates for beer and wine
- The paper uses the method pioneered by Angus Deaton that exploits spatial variation of beer and wine prices in Vietnam
- A limitation is that we do not provide elasticity estimates for spirits, another important alcohol type consumed in Vietnam
- A second limitation is that we do not provide cross-price elasticities for beer and wine in our analysis

For peer review only

For peer review only - http://bmjopen.bmj.com/site/about/guidelines.xhtml

INTRODUCTION

The use of alcohol in Vietnam measured in pure alcohol equivalent per person increased significantly from 3.8 litters in 2003-2005 to 6.6 litters in 2008-2010 (1). Excessive beer and wine consumption is not only harmful to health, it also increases domestic violence and traffic accidents. Approximately 33.7% of family violence cases in Vietnam are caused by the use of alcohol (2), while 36.2% and 0.7% of traffic accidents involving men and women were related to alcohol, respectively (3). About 36% of motorcyclists and 66.8% of car drivers involved in the 18,412 traffic accidents from July 2009 to October 2010 had higher than allowed level of blood alcohol concentration (4).

To reduce the harmful effects of the excessive use of alcohol, it is important to implement measures to reduce their consumption (5). Evidence from many countries demonstrates that tax increases resulting in higher prices are one of the most effective measures to control the excessive consumption of alcohol and the associated harms (6, 7). Gallet (2007) assessed the size of the price and income effects on alcohol demand by conducting a meta-analysis of 132 international studies and concluded that the price elasticities of beer and wine were about -0.83 and -1.11, respectively while the income elasticities were about 0.2 and 0.71, respectively (8). He concluded that the estimated responsiveness to price depends on the choice of demand functions, the model, the estimation method and the type of data used. He pointed out that nonlinear demand functions tend to generate smaller elasticities compared to linear demand functions. Bundit et al. (2013) summarized results of 12 studies estimating the demand for alcohol, including studies conducted in China, India, the Philippines, Taiwan and Thailand. The price elasticities of total alcohol ranged from - 0.02 in the Philippines to - 1.53 in China (9).

To assess the impact of tax policy on alcohol consumption in Vietnam, it is important to estimate price and income elasticities of alcohol demand, which indicate the level of consumers' sensitivity to price and income changes. In this paper we proxy for income using expenditure and therefore estimate expenditure elasticities of demand. Our study focuses on beer and wine, given that together they constitute much of the alcohol consumed in Vietnam. This study is the first to estimate elasticities for beer and wine in Vietnam using the Vietnam Household Living Standards Surveys (VHLSS) of 2010, 2012 and 2014.

For peer review only - http://bmjopen.bmj.com/site/about/guidelines.xhtml

METHOD

The description of the method given here borrows from Chelwa (2015) (10). We use data from the nationally representative Vietnam Household Living Standards Survey (VHLSS) for 2010, 2012 and 2014. The VHLSS is conducted by the General Statistics Office (GSO) in Vietnam and informs much of the country's planning processes. Each wave includes data on household income, expenditure and other household characteristics for 9,399 households in 3,133 clusters (or communes as they are known in Vietnam). A commune is the lowest administrative unit in Vietnam. We focus on the quantity and the monetary value of beer and wine bought in the previous month.

Given that we only have cross-sectional household data without prices and that the same alcohol tax is applied across Vietnam, we apply the Almost Ideal Demand System (AIDS) (11) and a unit value equation (11, 12) to estimate elasticities. The main identifying assumption is that prices vary at the cluster level, which in Vietnam is at the commune level. This assumption is reasonable given that households that are geographically proximate, i.e. in the same commune, face similar prices in Low and Middle Income countries (LMICs). This assumption generates exogenous variation in prices which is then used to estimate the elasticities.

Given that the VHLSS does not have price information, we proxy for prices using "unit values" calculated as:

$$v_{ic} = \frac{x_{ic}}{q_{ic}} \qquad (1)$$

Where v_{ic} ,x_{ic} and q_{ic} are respectively the unit value, expenditure and quantity of wine/beer by household *i* living in commune *c*. However, unit values are not the same thing as prices. They hide quality differences and are subject to measurement error. The method used in this paper (described in full below) addresses these shortcomings in using unit values in place of prices.

The method proceeds in a series of steps. First one conducts an Analysis of Variance (ANOVA) exercise to divide the total variation in unit values into "within commune

For peer review only - http://bmjopen.bmj.com/site/about/guidelines.xhtml

variation" and "between commune variation". The ANOVA is conducted to verify our main identifying assumption that prices, unit values in this case, vary at the commune level. A large F-statistic in this exercise leads to the conclusion that unit values vary across communes.

In a second step, one estimates "within commune" regressions of the form:

$$lnv_{ic} = \lambda + \beta lnX_{ic} + \boldsymbol{\gamma} \boldsymbol{Z_{ic}} + \psi ln\pi_c + e_{ic} \quad (2) \ \ and$$

$$w_{ic} = \alpha + \varepsilon lnX_{ic} + \boldsymbol{\delta} \boldsymbol{Z_{ic}} + \theta ln\pi_c + (FE_c + u_{ic}) \qquad (3)$$

Where w_{ic} is the share of wine/beer expenditure in total household expenditure for household i in commune c and lnv_{ic} is the log of the unit value, derived according to equation (1) for household i in commune c. lnX_{ic} is the log of total household monthly expenditure, $\boldsymbol{Z_{ic}}$ is a vector of household characteristics (household size, gender of household head, age of household head, years of schooling of the household head, employment status of the household head, urban/rural location of the household and ethnicity of the household head), FE_c is a commune fixed effect, and u_{ic} and e_{ic} are the standard regression error terms. We use characteristics of the household head as demographic control variables because household heads are known to influence the household's expenditure decisions. Given that the VHLSS does not contain price information, $ln\pi_c$ are the unobserved prices and consequently, equations (2) and (3) are estimated without them. However, the coefficients on the unobserved price terms can be recovered (see below).

A third step involves stripping the household level demand and unit values of the effects of household expenditure and household characteristics and then averaging across communes. The stripping and averaging is done according to the following equations:

$$\widehat{y_c^1} = \frac{1}{n_c}\sum_{i=1}^{n_c}(lnv_{ic} - \hat{\beta} lnx_{ic} - \hat{\boldsymbol{\gamma}} \boldsymbol{Z_{ic}}) \qquad (4)$$

For peer review only - http://bmjopen.bmj.com/site/about/guidelines.xhtml

and $$\widehat{y_c^2} = \frac{1}{n_c}\sum_{i=1}^{n_c}(w_{ic} - \hat{\varepsilon} ln x_{ic} - \hat{\boldsymbol{\delta}}\boldsymbol{Z}_{ic}) \qquad (5)$$

Where n_c is number of households in commune c, $\widehat{y_c^1}$ and $\widehat{y_c^2}$ are the estimates of, respectively, commune average unit value and commune average demand after removing the effects of household expenditure and household characteristics.

The next step involves regressing commune level demand, $\widehat{y_c^2}$, on commune level unit values, $\widehat{y_c^1}$. The coefficient on $\widehat{y_c^1}$ in such a regression can be obtained by dividing the covariance between $\widehat{y_c^2}$ and $\widehat{y_c^1}$ by the variance of $\widehat{y_c^1}$:

$$\hat{\phi} = \frac{Cov\left(\widehat{y_c^2}, \widehat{y_c^1}\right) - \frac{\widehat{\sigma^{12}}}{n_c}}{Var\left(\widehat{y_c^1}\right) - \frac{\widehat{\sigma^{11}}}{n_c^+}} \qquad (6)$$

where n_c^+ is the number of households in a commune reporting positive expenditures on tobacco and n_c is the number of households in a commune; $\widehat{\sigma^{12}}$ is the estimate of the covariance of the errors in equations (2) and (3); $\widehat{\sigma^{11}}$ is the variance of the errors in equation (2). The variance and covariance of the error terms correct for measurement error in unit values.

The final step applies quality correction formulas in obtaining the estimate of the price elasticity of demand, $\widehat{\varepsilon_P}$:

$$\widehat{\varepsilon_P} = \left(\frac{\hat{\theta}}{\overline{\overline{w}}}\right) - \hat{\psi} \qquad (7)$$

Where $\overline{w}$ is the average share of total household expenditure dedicated to wine/beer in the sample, and $\hat{\psi}$ and $\hat{\theta}$ are the estimates of the coefficients on the unobserved price terms in equations (2) and (3), respectively. $\hat{\psi}$ and $\hat{\theta}$ are recovered as follows:

$$\hat{\psi} = 1 - \frac{\hat{\beta}(\overline{w} - \hat{\theta})}{\hat{\varepsilon} + \overline{w}} \qquad (8) \; and$$

$$\hat{\theta} = \frac{\hat{\phi}}{1 + (\overline{w} - \hat{\phi})\hat{\zeta}} \qquad (9) \; with$$

For peer review only - http://bmjopen.bmj.com/site/about/guidelines.xhtml

For peer review only

$$\hat{\zeta} = \frac{\hat{\beta}}{\hat{\varepsilon} + \bar{w}(1 - \hat{\beta})} \qquad (10)$$

where $\hat{\beta}$ is the estimate of the coefficient on total household expenditure in equation (2), the within commune unit value equation, and $\hat{\varepsilon}$ is the coefficient on total household expenditure in equation (3), the within commune demand equation.

The expenditure elasticities of demand $\widehat{\varepsilon_e}$ is calculated according to Deaton (1990) (13):

$$\widehat{\varepsilon_e} = 1 + \left(\frac{\hat{\varepsilon}}{\bar{w}}\right) - \hat{\beta} \qquad (11)$$

We estimate elasticities separately for each VHLSS wave as well for the pooled sample. The standard errors for the elasticity estimates are obtained by the method of the bootstrap.

Table 1 reports some summary statistics for the variables used in the study. The average household budget shares allocated to wine and beer were respectively 4.5 % and 6.5 % over the period under study. The reported average unit values were VND17,400 (approximately USD0.80) for wine and VND19,700 (approximately USD0.92) for beer. VND stands for Vietnamese Dong, Vietnam's currency.

For peer review only

For peer review only - http://bmjopen.bmj.com/site/about/guidelines.xhtml

Table 1: Summary Statistics from the VHLSS

	Mean	*Std. Dev.*	*Mean*	*Std. Dev.*	*Mean*	*Std. Dev.*	*Mean*	*Std. Dev.*
	2010		2012		2014		2010-2014	
Average age of Household Head	48.3	14.2	49.7	14.2	50.7	14.1	49.6	14.2
Gender of Household Head (1 Male, 0 Female)	0.8	0.4	0.8	0.4	0.7	0.4	0.7	0.4
Average Household Size	3.9	1.6	3.9	1.6	3.8	1.6	3.9	1.6
Ethnicity of Household head (1- ethnic minority; 0-otherwise)	0.17	0.38	0.17	0.38	0.17	0.37	0.17	0.37
Average Year of schooling of Household head	7.58	4.40	7.60	4.40	7.74	4.41	7.64	4.41
Location of household (1 – urban, 0 - rural	0.28	0.45	0.29	0.45	0.30	0.46	0.29	0.45
Household head in wage employment (1- yes; 0- no)	0.41	0.49	0.39	0.49	0.40	0.49	0.40	0.49
Average wine expenditure (in thousands of VND)	38.4	56.4	46.7	61.5	51.1	48.8	45.2	56.3
Average beer expenditure (in thousands of VND)	98.7	111.9	117.4	108.6	140.5	133.9	118.1	119.6
Average unit value for wine (in thousands of VND)	14.2	31.1	18.2	34.7	20.1	15.2	17.4	28.8
Average unit value for beer (in thousands of VND)	16.1	10.2	19.9	9.7	23.6	12.0	19.7	11.1
Average household expenditure (in thousands of VND)	1,054.9	769.7	1,447.7	1,002.3	1,508.6	1,037.6	1,337.0	965.2
Share of total household expenditure dedicated to wine	0.049	0.062	0.042	0.048	0.044	0.053	0.045	0.055
Share of total household expenditure dedicated to beer	0.068	0.062	0.060	0.057	0.067	0.056	0.065	0.058

For peer review only

For peer review only - http://bmjopen.bmj.com/site/about/guidelines.xhtml

Source: Calculated from VHLSS. VND stands for Vietnamese Dong.

Patients and public involvement

Patients and the public were not involved in this study.

RESULTS

The results of the ANOVA exercise are discussed here but not reported for reasons of space. They are, however, available from the authors upon request. The R squared from the ANOVA exercise indicates that at least 85% of the variation in unit values for wine and beer is attributable to variations between communes. The F statistics and the associated p values allow us to reject the null hypothesis of no spatial variation in 2010, 2012, 2014 and pooled data. In other words, prices vary significantly between communes in Vietnam.

The results of the unit value regressions (equation 2) are reported in Table 2. The results show that households with higher expenditure report higher unit values. In other words, unit values reflect choices about quality. These so-called expenditure elasticities of quality range between 0.06 and 0.07 for wine and between 0.22 and 0.32 for beer. This means that the reported unit values of wine and beer rise by 0.6% to 0.7% and by 2.2% to 3.2% for every 10% increase in household expenditure, respectively.

Households with older household heads and with heads with more schooling report higher unit values for wine. The impact of household size is negative for both wine and beer, implying that larger households report lower unit values. Households living in urban areas tend to have higher unit values for both alcohol products while ethnic minorities report lower unit values for wine.

For peer review only - http://bmjopen.bmj.com/site/about/guidelines.xhtml

Table 2: Wine and Beer Unit Values Coefficients

	Wine				Beer			
	2010	2012	2014	Pooled	2010	2012	2014	Pooled
VARIABLES	lnunit_v7				lnunit_v8			
LnX	0.060***	0.070***	0.066***	0.137***	0.316***	0.238***	0.213***	0.343***
	(0.009)	(0.010)	(0.011)	(0.006)	(0.021)	(0.022)	(0.021)	(0.012)
Gender	-0.025	-0.018	0.005	-0.003	-0.015	-0.001	0.011	0.008
	(0.016)	(0.016)	(0.018)	(0.010)	(0.027)	(0.027)	(0.026)	(0.016)
LnAge	0.030	0.131***	0.082***	0.118***	-0.066	-0.069	-0.095**	-0.048*
	(0.021)	(0.023)	(0.026)	(0.014)	(0.042)	(0.045)	(0.045)	(0.026)
LnSchool	0.111***	0.155***	0.164***	0.133***	-0.003	-0.008	-0.006	-0.027**
	(0.009)	(0.010)	(0.011)	(0.006)	(0.023)	(0.022)	(0.020)	(0.013)
Employment	0.005	0.025*	-0.002	0.015*	-0.027	-0.014	-0.044*	-0.019
	(0.012)	(0.013)	(0.013)	(0.008)	(0.025)	(0.026)	(0.025)	(0.015)
Ln Size	-0.030**	-0.064***	-0.025	-0.093***	-0.192***	-0.183***	-0.142***	-0.224***
	(0.015)	(0.016)	(0.017)	(0.010)	(0.032)	(0.031)	(0.031)	(0.019)
Urban	0.117***	0.109***	0.137***	0.096***	0.053**	0.115***	0.128***	0.074***
	(0.013)	(0.014)	(0.015)	(0.009)	(0.024)	(0.023)	(0.022)	(0.014)
Ethnic	-0.028*	-0.023	-0.053***	0.011	0.076*	0.050	0.029	0.055**
	(0.015)	(0.015)	(0.017)	(0.010)	(0.040)	(0.044)	(0.048)	(0.026)
Constant	1.851***	1.564***	1.808***	1.170***	0.909***	1.586***	1.971***	0.836***
	(0.102)	(0.114)	(0.129)	(0.069)	(0.215)	(0.236)	(0.242)	(0.134)
Observations	4,241	4,199	3,758	12,198	1,918	1,717	1,705	5,340
R-squared	0.112	0.138	0.151	0.149	0.156	0.120	0.109	0.179

Robust standard errors in parenthesis. LnX (natural logarithm of household expenditure), Gender (gender of household head), LnAge (natural logarithm of age of household head), LnSchool (natural logarithm of years of schooling of household head), Employment (employment status of household head), Urban (location of household), Ethnic (ethnic group of household head). *** p<0.01, ** p<0.05, * p<0.1

Results of regressing wine and beer budget shares on household expenditure and other household characteristics (equation 3) are reported in Table 3. The results show that there is a negative and statistically significant relationship between household expenditure and the share of the household budget allocated to wine and beer. More years of schooling among household heads is related to a smaller budget share on both wine and beer.

For peer review only - http://bmjopen.bmj.com/site/about/guidelines.xhtml

Households with a male head, located in urban areas and from an ethnic minority devote larger budget shares to wine.

Table 3: Beer and Wine Budget Shares

	Wine				Beer			
	2010	2012	2014	Pooled	2010	2012	2014	Pooled
VARIABLES	w_v7				w_v8			
Lnx	-0.037***	-0.027***	-0.038***	-0.032***	-0.013***	-0.023***	-0.018***	-0.017***
	(0.004)	(0.002)	(0.004)	(0.002)	(0.005)	(0.007)	(0.004)	(0.003)
Sex	0.002	0.004**	0.006***	0.004***	0.004	0.003	0.002	0.003*
	(0.003)	(0.002)	(0.002)	(0.001)	(0.004)	(0.003)	(0.003)	(0.002)
Lnage	-0.009***	-0.002	0.001	-0.003	-0.011	-0.006	-0.007	-0.007*
	(0.003)	(0.003)	(0.004)	(0.002)	(0.007)	(0.006)	(0.006)	(0.004)
Lnschool	-0.001	-0.005***	-0.001	-0.002***	-0.005	-0.013***	-0.012***	-0.011***
	(0.001)	(0.001)	(0.001)	(0.001)	(0.003)	(0.003)	(0.003)	(0.002)
EmW	-0.009***	-0.003*	-0.006***	-0.006***	-0.002	-0.000	-0.000	-0.000
	(0.002)	(0.001)	(0.002)	(0.001)	(0.004)	(0.003)	(0.003)	(0.002)
EmSelf	-0.006***	-0.000	-0.002	-0.003***	0.000	0.002	-0.001	0.000
	(0.002)	(0.001)	(0.002)	(0.001)	(0.004)	(0.004)	(0.003)	(0.002)
Lnsize	0.003	-0.003	0.009*	0.002	-0.022***	-0.012**	-0.016***	-0.018***
	(0.003)	(0.003)	(0.004)	(0.002)	(0.006)	(0.005)	(0.005)	(0.003)
Urban	0.004**	0.005***	0.007***	0.004***	0.001	0.008	-0.003	0.002
	(0.002)	(0.002)	(0.002)	(0.001)	(0.004)	(0.005)	(0.003)	(0.003)
Ethnic	0.018***	0.019***	0.018***	0.019***	0.006	0.009	-0.009	0.003
	(0.002)	(0.002)	(0.002)	(0.002)	(0.006)	(0.006)	(0.006)	(0.004)
Constant	0.326***	0.244***	0.292***	0.274***	0.240***	0.296***	0.281***	0.261***
	(0.029)	(0.019)	(0.028)	(0.015)	(0.048)	(0.051)	(0.038)	(0.027)
Observations	4,241	4,199	3,758	12,198	1,918	1,717	1,705	5,340
R-squared	0.253	0.236	0.270	0.247	0.047	0.086	0.086	0.066

Robust standard errors in parenthesis. LnX (natural logarithm of household expenditure), Gender (gender of household head), LnAge (natural logarithm of age of household head), LnSchool (natural logarithm of years of schooling of household head), Employment (employment status of household head), Urban (location of household) and Ethnic (ethnic group of household head). *** p<0.01, ** p<0.05, * p<0.1

The price elasticity estimates, $\widehat{\varepsilon_P}$, for 2010, 2012, 2014 and the pooled sample are presented in Table 4.

For peer review only - http://bmjopen.bmj.com/site/about/guidelines.xhtml

For peer review only

Table 4: Estimates of Price elasticity of Demand for Wine and Beer

	Observed Coef.	Bootstrap Std. Err.	Z	P>z	Normal-based [95% Conf. Interval]	
**Wine**						
2010	-0.237	0.085	-2.800	0.005	-0.403	-0.071
2012	-0.491	0.029	-16.950	0.000	-0.548	-0.434
2014	-0.359	1.409	-0.260	0.799	-3.122	2.403
Pooled	-0.317	0.030	-10.560	0.000	-0.376	-0.258
**Beer**						
2010	-0.251	0.025	-10.140	0.000	-0.300	-0.203
2012	-0.305	0.032	-9.500	0.000	-0.368	-0.242
2014	-0.346	0.029	-11.850	0.000	-0.404	-0.289
Pooled	-0.283	0.014	-20.120	0.000	-0.311	-0.256

Source: Calculated from VHLSS

All estimates are statistically significant at the 1% level except for wine in 2014. The price elasticity of demand for wine ranges from -0.237 to -0.491 with the pooled estimate centering on -0.317. This means that the demand for wine in Vietnam is expected to decline on average by 3.17% for every 10% rise in wine prices. The price elasticity of demand for beer ranges from -0.251 to -0.346 with the pooled estimate centering on -0.283. This means that the demand for beer is expected to decline by 2.83% on average for every 10% rise in beer prices.

The estimates of the expenditure elasticities, $\hat{\varepsilon_e}$, for 2010, 2012, 2014 are reported in Table 5. As in Table 4, all estimates are statistically significant at the 1% level except for wine in 2014. For the pooled data, the expenditure elasticities are 0.156 and 0.401 for wine and beer, respectively. A 10% increase in expenditure leads to a 1.6% and 4.0% increase, on average, in the demand for wine and beer, respectively. This shows that increases in household income, proxied here by household expenditure, will increase wine and beer consumption in Vietnam.

For peer review only - http://bmjopen.bmj.com/site/about/guidelines.xhtml

Table 5. Estimates of the expenditure elasticity of demand

	Observed Coef.	Bootstrap Std. Err.	z	P>z	Normal-based [95% Conf. Interval]	
			**Wine**			
2010	0.122	0.065	1.890	0.059	-0.005	0.249
2012	0.339	0.040	8.550	0.000	0.261	0.416
2014	0.097	0.080	1.210	0.225	-0.060	0.255
Pooled	0.156	0.034	4.630	0.000	0.090	0.222
			**Beer**			
2010	0.484	0.060	8.000	0.000	0.365	0.602
2012	0.406	0.101	4.000	0.000	0.207	0.604
2014	0.503	0.053	9.420	0.000	0.398	0.607
Pooled	0.401	0.036	11.110	0.000	0.330	0.472

Source: Calculated from VHLSS

DISCUSSION

This paper estimates the first ever price and expenditure elasticities of demand for wine and beer in Vietnam using the VHLSS. The statistical analysis revealed that wine and beer in Vietnam are price inelastic. In other words, the quantity demanded of wine and beer responds less than proportionately to price increases. These results are in line with elasticity estimates from other parts of the world (8).

Our results on the price inelastic nature of the demand for wine and beer has two immediate implications for the authorities in Vietnam. First, it means that tax measures can be used as an effective tool to control the excessive consumption of alcohol in Vietnam. Second, the government can raise additional tax revenue by increasing the level of alcohol taxes in the country.

The results on the expenditure elasticities of demand also have implications for the country. Our estimated elasticities of demand, which are all positive, mean that the quantity demanded of wine and beer in Vietnam increases every time household

For peer review only - http://bmjopen.bmj.com/site/about/guidelines.xhtml

expenditure (or household income) increases. This means that alcohol consumption will continue to increase, and increase at a fast pace, given that Vietnam is a fast growing economy. The gives further impetus to the use of tax measures in curbing excessive demand especially in an environment of fast rising incomes.

Our paper has three limitations, however. First, we are unable to provide elasticity estimates for spirits, another important alcohol category. This is because we do not have good data on spirits to enable us estimate their elasticities. Second, we are unable to provide cross-price elasticities between wine and beer. Cross price elasticities are important because policymakers often want to know how the demand for one good responds to levying a tax on a close substitute. Lastly, our elasticity estimates are conditional elasticity estimates and not total elasticity estimates. In other words, we only provide demand responses for current drinkers of wine and beer and not for the entire population. In other words, we do not estimate total price elasticities. However, empirically, conditional elasticities are often half the absolute value of total elasticities (14). This means that the total demand response to prices is likely to be much higher than the estimates presented in this paper.

For peer review only

Contributors:

PNT did the analysis with the help of GC. NTTH, HR, LTT and PTHA helped with the writing of the manuscript.

Funding:

This work was supported by the International Development Research Centre grant number 107200-002.

Ethics approval: This study did not require ethics approval as it uses the Vietnam Household Living Standards Survey which is a secondary dataset that is publically available

Competing Interests: None

Provenance and peer review: Not commissioned; externally peer reviewed

Data sharing statement: All datasets are publicly available

For peer review only - http://bmjopen.bmj.com/site/about/guidelines.xhtml

References

1. Ministry of Industry and Trade (MOIT). The evaluation report for the construction industry development planning: Beer and Wine Beverage in Vietnam to 2015, vision 2025. 2008.
2. General Statistics Office. Vietnam National Survey of Family Violence. 2010.
3. World Health Organization. Global status report on alcohol and health 2014. Geneva 2014.
4. World Health Organization, Health Environment Management Survey in Vietnam: 2009- 2010. 2010.
5. Edwards, G. PA, T. F. Babor, S. Casswell, R. Ferrence, N. Giesbrecht, et al. Alcohol Policy and the Public Good. Oxford: Oxford University Press. 1994.
6. Anderson P, Chisholm D, Fuhr D. Effectiveness and cost-effectiveness of policies and programmes to reduce the harm caused by alcohol. Lancet. 2009;373:2234–46.
7. Babor T, Caetano R, Casswell S, Edwards G, Giesbrecht N, Graham K, et al. Alcohol: No ordinary commodity. Research and public policy (2nd ed.). Oxford, England: Oxford University Press.: 2010.
8. Craig A, Gallet. The demand for alcohol: a meta-analysis of elasticities. The Australian Journal of Agricultural and Resource Economics. 2007;51:121-35.
9. Bundit S., Kevin S., Joanna C., Robert S., Jürgen R. Elasticity of alcohol consumption, alcohol-related harms, and drinking initiation in low- and middle-income countries: A systematic review and meta-analysis. International Journal of Alcohol and Drug Research. 2013;2(1):45-58.
10. Chelwa G. The economics of tobacco control in some African countries 2015. PhD Thesis, The University of Cape Town.
11. Deaton A. S., J. Muellbauer. An Almost Ideal Demand System. American Economics Review. 1980a;70:312-26.
12. S.J.Praisand, H.S.Houthakker. The Analysis of Family Budget. Cambridge University Press Cambridge 1955.
13. Deaton. Price Elasticity From Survey Data: Extensions and Indonesian Results. Journal of Econometrics. 1990.

For peer review only - http://bmjopen.bmj.com/site/about/guidelines.xhtml

14. International Agency for Research on Cancer. Handbook on the effectiveness of tax and price policies for tobacco control. Geneva: World Health Organization 2011
For peer review only

For peer review only - http://bmjopen.bmj.com/site/about/guidelines.xhtml
